# Does the human immune system ever really become “senescent”?

**DOI:** 10.12688/f1000research.11297.1

**Published:** 2017-08-04

**Authors:** Graham Pawelec

**Affiliations:** 1 Health Sciences North Research Institute, Sudbury, ON, Canada; 2 Division of Cancer Studies, King’s College London, London, UK; 3 The John van Geest Cancer Research Centre, School of Science and Technology, Nottingham Trent University, Nottingham, UK; 4 Second Department of Internal Medicine, University of Tübingen, Tübingen, Germany

**Keywords:** immune aging, immunoscenecene, immune system, immune cell distribution, inflammaging

## Abstract

Like all somatic tissues, the human immune system changes with age. This is believed to result in an increased frequency of, and susceptibility to, infectious disease and to contribute to a wide range of non-communicable age-associated diseases in later life, especially cancer, cardiovascular disease, and autoimmunity. The majority of studies addressing immune ageing has been cross-sectional, but limited longitudinal studies are contributing to a better understanding of age-associated changes, as opposed to differences, and their clinical relevance. However, intriguing differences are emerging that implicate highly context-dependent immune ageing processes, mitigating against current generalisations concerning human immunosenescence and indicating the necessity for detailed comparisons of different populations, even those that would appear quite similar at first glance.

## Introduction

As reflected in numberless papers, it is commonly accepted as received wisdom that “declining function of the immune system, termed "immunosenescence", leads to a higher incidence of infection, cancer, and autoimmune disease related mortalities in the elderly population” ^1^, encouraging a great deal of effort to prevent or reverse this state ^2, 3^. Here, limiting the discussion to humans and focusing on adaptive immunity, I reconsider the veracity of the above assertion in light of data published over the last 3 years and in the face of a certain degree of confusion in the literature, which may be perplexing and misleading to the uninitiated.

First, it is necessary to clearly state what we think we mean by the term “immunosenescence”, which tends to mean different things to different people. It is often assumed, especially by non-immunologists, that this term refers to the cell biological concept of “replicative senescence”, whereby somatic human cells cease dividing at the “Hayflick limit” after a finite number of cell divisions owing to telomere attrition ^4, 5^. However, this is not what immunologists generally mean by “immunosenescence”, as illustrated by reference 1 above. Moreover, in my view, this term should be reserved for a state defined by robust measures of immune parameters (biomarkers) that are different in younger and older individuals and which have been associated with a clearly detrimental clinical outcome (e.g. mortality, frailty, poor response to vaccination, etc.). Here it should be noted that many studies report differences between older and younger people without being able to associate these directly with clinical outcome. The associations that are made with clinical outcome most commonly distinguish between immune biomarkers in older populations of similar ages, which are also different from those in the young. Hence, they should not be referred to as age-associated changes but only as age-associated differences because the comparisons between younger and older are mostly based on cross-sectional studies. It is an assumption that these differences represent changes that would occur in the younger population given sufficient time. This assumption is unlikely to be correct, given the markedly different circumstances of older people born in the early to middle 20 ^th^ century and young people born towards the end of that century. These differences are essentially impossible to control for and are likely to include genetic, nutritional, environmental, psychosocial, and educational factors as well as many others. What mostly remains possible in human studies is to perform longitudinal follow-up on people already advanced in years, as in cohorts such as the Leiden 85-Plus, which already initiated some immunological studies nearly 20 years ago ^6^. At least in such selected populations, one can determine immune parameters at baseline, and their changes over time, that can be associated with clinical outcome within a feasible time-frame. These could then be tested for their applicability in younger subjects.

Notwithstanding the above considerations, much useful information can of course be garnered from cross-sectional studies establishing differences in immune biomarkers in older adults and determining which are associated with properties assigned to a person who is “immunosenescent”, i.e. infection, cancer, cardiovascular disease, autoimmunity (and response to vaccination), etc. A comparison of clinically relevant differences and similarities between younger and older populations, in agreement or disagreement with immune biomarkers found to predict certain clinical outcomes in longitudinal studies of the elderly, will help to define the lowest common denominator likely to be globally relevant to these health outcomes. Thus, of all the myriad of age-associated factors reported to be different in younger and older people, and the far fewer parameters also found to be informative in longitudinal studies, it may be possible to select those that are crucial, which could provide information on mechanisms and offer opportunities for rational intervention. Now the critical question becomes do we have any idea what these immune parameters are?

## Characteristics of human immune ageing

Of many disparate findings in the literature, one immune parameter stands out as universally different in younger and older adults, consistently reported in a multitude of studies. This is the finding that older adults possess vanishingly low numbers and percentages of naïve CD8 ^+^ T cells in their peripheral blood, as first reported many years ago, for example 7. Reciprocally, as might be expected from the principle that antigen-stimulated naïve cells differentiate into effector and memory cells, the numbers and proportions of memory CD8 ^+^ T cells were reported to be higher in the elderly in that same study ^7^. However, unlike the findings on naïve CD8 ^+^ T cells, higher CD8 ^+^ memory cells in the elderly are not universally reported. In the meantime, the reason for this is well established, although still not understood—namely, that accumulations of late-stage memory cells are seen in older people infected with human herpesvirus 5 (HHV5 or cytomegalovirus [CMV]) but not other herpesviruses ^8– 10^, as confirmed in a recent systematic review ^11^. Because CMV seroconversion increases with age ^12^ and is affected by socioeconomic factors ^13^, it confounds the effect of age on immune parameters, as was also seen in the systematic review ^11^. It is important to note that these results have been reproduced not only in Western populations, which are the most commonly studied but not necessarily the most representative human population, but also in people living in less-industrialized countries. There is some evidence that this loss of CD8 ^+^ naïve cells occurs at an earlier chronological age in low- and middle-income countries ^14^, possibly due at least partly, if not only, to greater exposure to pathogens driving the naïve CD8 ^+^ T cells to differentiate to effector and memory cells in these populations, something that is harder to detect in Western societies. This provides enduring protection against pathogens encountered earlier in life but may reduce the naïve T cell repertoire for *de novo* challenges in later life and contribute to the greater susceptibility of the elderly to emerging pathogens. It is also important to note that in those rare studies that have considered sex differences, the markedly lower levels of CD8 ^+^ naïve T cells have been found in both women and men, as for example in our study of Berliners ^15^. Differences between younger and older adults are seen to a lesser extent for CD4 ^+^ naïve T cells, and for B cells, as well as for some elements of innate immunity, especially dendritic cells (DCs) and neutrophils ^16, 17^, but all immune cells are affected to some degree. In all instances, it must be borne in mind that tissue compartments other than peripheral blood may not, or most likely do not, exhibit the same patterns of cell subset distribution ^18^. Thus far, the data on immune cell distribution in tissues other than blood in humans are too sparse for any clinical correlates to be established.

## Impact on vaccination

Because it is the CD8 ^+^ T cells that are predominantly acting as cytolytic effectors eliminating virally infected cells, the CD4 ^+^ T cells that help B cells to produce neutralising antibodies, and DCs that present antigen to the T cells, one might expect that a paucity of such cells could result in higher susceptibility to viral infections in old age. There are some data on this question in the context of vaccination against infectious agents, where the major public health interest in immunosenescence lies. While most studies have focussed on seasonal influenza, the question of whether lower numbers of naïve T cells really compromise immune responses has been relatively rarely addressed because it is necessary to study responses to pathogens to which the person has definitely not been previously exposed. Vaccinating volunteers with yellow fever (YF) vaccine does provide such an opportunity, and such studies confirm that differences in the CD4 ^+^ and CD8 ^+^ T cell response, and in DC function, are demonstrable in older adults with poorer responses relative to the young ^19^. Amongst other parameters, this study quantified recent thymic emigrants as a measure of naïve T cells and demonstrated that their paucity indeed correlated with poorer responses to a new pathogen (i.e. the vaccine). This is consistent with the long-held belief that thymic involution early in life contributes decisively to the very small numbers of naïve T cells in the elderly ^20^. Although it is not perfectly clear why thymic involution occurs, it is hypothesized that the requirement for maximising immune defence potential is greatest during childhood to prevent death from infection ^21^. After puberty and reproductive age, exposure to new pathogens is less likely and immune memory for local pathogens is paramount. In many documented cases, we know that immune memory can be very long-lasting, so resources are concentrated on maintaining defence against tangible pathogens rather than investing resources in the potentially hazardous (due to possible errors in negative selection leading to autoimmunity) and certainly energy-intensive production of new naïve T cells that will most likely never be needed. Again, relatively few studies have been performed to test this, but, in the case of YF vaccination as described above, a different group of investigators demonstrated the presence of “stem-cell-like” CD8 memory cells in YF vaccinees up to 25 years later ^22^. Owing to these stem-cell-like self-renewal properties, memory can be maintained perhaps over the lifetime; ageing of these cells is thus not necessarily a problem. Nonetheless, and for largely unknown reasons, adaptive immunological memory to a wide range of different pathogens (as reflected in the duration of protection after successful vaccination) is extremely variable. It may last a lifetime (e.g. measles) but it may last only half a decade (e.g. pertussis) and all times in between (see, for example, www.immune.org.nz/vaccines/efficiency-effectiveness). In the case of persistent pathogens such as VZV and TB, waning of immune control with age can result in reactivation and clinically relevant disease manifestations. But despite waning of memory for these pathogens, protection is maintained for decades in the majority of cases, so the main problem remains the paucity of naïve cells in older people who have survived for a much longer period of time than would mostly be expected to be the case “in the wild”. For these people, exposures to neoantigens might be hazardous if they happened not to have any remaining antigen-specific T cells in their shrunken repertoire. This may explain, for example, why older people suffered a very much higher mortality rate than younger people during the SARS epidemic, but this was not demonstrated at the time ^23^.

The impact of immunosenescence on influenza vaccination is much more complicated to dissect because of the ever-changing balance of seasonal strains and indeterminate degree of T cell memory. Seasonal vaccines seek to stimulate the production of neutralising antibodies or increase the level already present but are not directed at stimulating T cell responses that may be critical for clinical protection. The way in which the antibody “response” is assigned as “responder” or “non-responder” is in itself problematic because the presence of an already-high titre of antibody that is not further increased by vaccination can result in classification as a “non-responder”. This alone may help to account for discrepancies in the literature as to differences in the fraction of older people versus younger people who do respond to the vaccine. Indeed, one recent study taking vaccination history into account concluded that from the point of view of antibody titre, older people did in fact respond as well as younger people ^24^. However, protection against infection requires the integrity of T-cell-mediated responses, and here data show that a lower proportion of elderly than younger people may be capable of mounting a T cell response, especially if they are infected with CMV, as many older people are ^25^, and that they may not respond as well as the young ^26^. In this context, we may approach a definition of the harm that immunosenescence can do rather than acting merely as a variety of immune deficiency. Thus, evidence is emerging that CD8 ^+^ memory cells in elderly people may behave in a pathogenic manner to a greater degree than in the young. A suggested mechanism for this is based on the recent finding that at least some late-stage differentiated CD8 ^+^ T cells, characterised by a lack of expression of the costimulatory receptors CD27 and CD28, with short telomeres and with little clonal expansion capacity, and expressing receptors such as HNK-1, shared with natural killer (NK) cells, otherwise known as CD57 (commonly referred to as “senescent cells” in the literature), may indeed be malfunctional. This has been established for NK cells and is mediated by the inappropriate release of granzyme ^27^, which causes tissue damage and inflammation ^28^. There is preliminary evidence that CD8 ^+^ T cells can behave in a similar manner and contribute to “inflammaging” ^29^.

## What is “inflammaging” as opposed to “immunosenescence”?

It is often considered that the state of slightly raised inflammatory mediators commonly seen in elderly people, some of which are associated with frailty and mortality ^30^ (hence dubbed “inflammaging” ^31^) is part of the state referred to as “immunosenescence”. However, the tissue of origin of the pro-inflammatory mediators is, to a great extent, unclear, and it is likely that the majority is not derived from immune cells but rather from other, possibly replicatively senescent, cells ^32^. Indeed, in early studies in a very elderly Swedish population, we found that 2-, 4-, and 6-year mortality at follow-up was weakly associated with a cluster of immune parameters including higher levels of late-stage differentiated CD8 ^+^CD28 ^–^ T cells and lower levels of B cells but was more strongly associated with higher levels of IL-6 together with cognitive impairment. These two clusters were independent of each other and were at least additive in their effects on mortality ^33^. Similar findings on these variables as independent mortality risk factors have sometimes been reported from some other studies in different countries, e.g. the Spanish CARRERITAS study ^34^. Therefore, in my opinion, the terms “inflammaging” and “immunosenescence” should also be used independently of one another. It is also noteworthy that the “immune risk profile”, or IRP as we called it ^35^, did not include the numbers or percentages of naïve CD8 ^+^ T cells, suggesting that in this very elderly population probably no longer exposed to many novel pathogens, it was not important to survival to maintain an extensive naïve T cell repertoire. In fact, in studies in other populations, a disadvantage was found to accrue in individuals with higher proportions of naïve cells (see below).

## Context-dependency of the factors defining “immunosenescence”

According to the definition given at the beginning, senescence has to be defined in terms of known detrimental clinical associations. The fact that one of the main surrogate markers of inflammaging, IL-6 (together with cognitive impairment), correlated better with mortality than did the IRP suggests that many of the unequivocal negative health effects associated with old age and attributed to immunosenescence actually have little to do with immunosenescence *per se*. As discussed above, the main differences between younger and older people reside in the different distribution of naïve and memory elements of the adaptive immune system (both T and B cells, the latter not discussed in detail here for reasons of space constraints) and more subtle differences in innate immunity. The first line of defence against pathogens is usually the neutrophil, and in older people neutrophil function may be compromised relative to the young ^36^. Neutrophils have been included in clusters of larger numbers of immune and non-immune parameters, many related to inflammation, that are associated with mortality ^37^. Such clusters of parameters may be informative for ageing trajectories, even at a relatively young age. Thus, an influential study by Belsky *et al*. ^38^ followed nearly 1,000 young people (26 years of age) in New Zealand for 12 years and measured a panel of biomarkers that they then correlated with relative physical and cognitive performance and appearance. Even at the age of only 38, some individuals were biologically more aged than others, and this correlated with clusters of markers including CRP, white blood cell count, and leucocyte telomere length. Extending such studies in future to include more granular and discriminatory immune biomarkers will allow us to determine whether there really are any biomarkers of immunosenescence in the strict sense of mediating a direct association with detrimental clinical outcomes or whether the differences in immune parameters observed in older people relative to young people mostly or entirely reflect adaptive responses to the immunological history and current situation of the individual. Where limited knowledge of the relevance of immune parameters like the IRP for predicting survival does exist, it appears that there are notable differences even between populations that one might *a priori* expect to be quite similar, such as Swedish, Dutch, and Belgian. However, different birth cohorts separated by decades, coupled with nutritional variation, associated differences in the gut microbiota and pathogen exposures (including CMV), socioeconomic circumstances, and any number of other variables could explain why these differences arise. Hence, at this stage, we should resist generalising the outcomes associated even with the same simplest biomarkers of “immunosenescence” in different populations. This is most strikingly illustrated by our findings in the Belgian population as opposed to the Swedish, where a CD4:8 ratio of <1 and CMV-seropositivity indicated a risk profile in the latter, but exactly this combination was associated with better survival in the former ^39^. Even more strikingly, associations with survival in the Belgian study were seen exclusively in women, with absolutely no differences in men ^39^. A deeper analysis at the single-cell level using more advanced techniques such as CyTOF, RNA-Seq, Nanostring, and others should soon begin to provide a more detailed view of the relevant differences. Nonetheless, the above considerations will still apply to ever-richer datasets and will need to be borne in mind when interpreting these studies.

## Conclusions

Biomarkers of immune ageing as established in cross-sectional studies unequivocally document multiple differences between younger and older populations. Some markers distinct to older people can be associated with important health parameters such as frailty and responses to vaccination, but these and especially factors associated with mortality must be viewed in the context of the particular population in which they were established. Truly universal age-associated changes in immune markers mostly seem limited to the reduction in numbers and proportions of peripheral blood naïve T cells due to thymic involution and possibly to dysfunctional short-lived innate immune cells, reflecting ageing of the haematopoietic stem cell system ^40^ and the poorly defined detrimental systemic milieu in older animals ^41^. Hence, my answer to the question posed in the title is yes, in my opinion the human immune system does undergo senescence, and this is predominantly as a result of increasing holes in the naïve T cell repertoire and creeping exhaustion and malfunction of some of the cells responsible for immune memory of certain pathogens. The latter phenomenon, together with changes to haematopoiesis and innate immunity, contributes to the enhanced inflammatory status and tissue damage associated with “inflammaging”.

## Abbreviations

CMV, cytomegalovirus; DC, dendritic cell; IRP, immune risk profile; NK, natural killer; YF, yellow fever.
